# Microsurgical resection of anterior clinoidal meningiomas with arterial engulfment: lessons from 2 contrasting cases

**DOI:** 10.3171/2025.10.FOCVID25169

**Published:** 2026-01-01

**Authors:** Umid Sulaimanov, Ufuk Erginoglu, Abdullah Keles, Yerkebulan Serikkanov, Franco Vera Figueroa, Umut Tan Sevgi, Oyku Ozturk, Mustafa K. Baskaya

**Affiliations:** Department of Neurological Surgery, School of Medicine and Public Health, University of Wisconsin, Madison, Wisconsin

**Keywords:** anterior choroidal artery, clinoidal meningioma, intraoperative complication, neurophysiological monitoring, skull base surgery, vascular encasement

## Abstract

Anterior clinoidal meningiomas are among the most complex skull base tumors because of their proximity to the optic apparatus, internal carotid artery (ICA), and its branches. When vessels are engulfed or encased, the difficulty of resection and the risk to the vasculature increase significantly. Surgical management requires balancing maximal resection with neurovascular preservation. The authors present 2 cases with ICA engulfment. The first involved anterior choroidal artery injury managed using collateral assessment, neurophysiological monitoring, and indocyanine green angiography as a surrogate intraoperative balloon test occlusion. The second emphasizes meticulous dissection and progressive debulking to achieve gross-total resection while preserving neurological function.

The video can be found here: https://stream.cadmore.media/r10.3171/2025.10.FOCVID25169

## Transcript

In this video, we present 2 cases of anterior clinoidal meningiomas with extensive involvement of the supraclinoid internal carotid artery (ICA) and its branches, as well as adjacent neurovascular structures, both achieving gross-total resection via pterional microsurgical resection with extradural clinoidectomy and optic canal unroofing. Intraoperative neuromonitoring and indocyanine green angiography guided meticulous tumor-vessel dissection. The first case demonstrates management of an anterior choroidal artery (AChA) injury, while the second highlights strategies to prevent vascular injury during safe resection.

### 0:48 Background Information.

Clinoidal meningiomas are challenging due to their proximity to critical neurovascular structures, including the optic apparatus, ICA, and branches. Vascular complications occur in 1%–19% of anterior skull base meningiomas, often linked to arterial encasement of supraclinoid ICA, M1, A1, and larger postoperative infarction volumes.^1^ Tumor origin, extension, and the presence of protective arachnoid membranes affect injury risk, with vessel narrowing suggesting wall involvement and excessive traction raising rupture risk, underscoring the importance of meticulous microsurgical dissection.^2–4^

### 1:20 Case 1 Presentation.

The first case is a 50-year-old woman with an incidentally discovered large left clinoidal meningioma, normal ophthalmological findings, and no neurological deficits, referred to our center for further management.

### 1:30 Preoperative Imaging.

Magnetic resonance imaging (MRI) showed a 3.5-cm left anterior clinoidal meningioma compressing the temporal lobe, abutting the optic apparatus, and CT angiogram (CTA) further revealed engulfing the supraclinoid ICA with possible AChA and posterior communicating artery (PComA) involvement, mild M1 narrowing, diminutive A1, and preserved anterior circulation.

### 1:48 Management.

Clinoidal meningiomas arise from the anterior clinoid dura, adjacent to the optic nerve, ICA, oculomotor nerve, and cavernous sinus. Their dural and arachnoid origin, described by Al-Mefty, defines surgical difficulty and risk of vascular or optic involvement.^2^ Standard management is microsurgical resection via a pterional transsylvian approach with intra- or extradural anterior clinoidectomy and optic canal unroofing for ICA and optic nerve exposure.^5^ Alternative routes include cranio-orbital (modified orbitozygomatic approach), lateral supraorbital, or modified approaches, while subtotal resection with radiosurgery is reserved for arterial encasement with intraluminal involvement.

### 2:22 Patient Positioning and Skin Incision.

Under general anesthesia, the patient was placed supine with the head turned right, and a left pterional craniotomy with extradural anterior clinoidectomy and optic canal unroofing was performed. For large anterior clinoidal meningiomas, we favor this approach as it improves intradural exposure, allows for proximal ICA control in its clinoidal segment, and facilitates the removal of the involved dura of the clinoid and optic canal. After the craniotomy, the dura of the anterior skull base and temporal lobe was elevated extradurally to expose the orbital roof, anterior clinoid process, superior orbital fissure, and V1–V2 of the trigeminal nerve, enabling safer clinoidectomy and optic unroofing. The meningo-orbital band was dissected and incised, mobilizing the dura propria from the cavernous sinus lateral wall. The anterior clinoid process has been then exposed, the orbital roof thinned with a small orbitotomy, followed by optic canal unroofing and stepwise drilling and microrongeuring of the clinoid.

### 3:12 Dural Opening and Arachnoid Dissection.

The dura was opened curvilinearly, exposing the sylvian fissure. The distal arachnoid was dissected to identify M2 branches, and careful planes were developed between the tumor and the brain. Early distal control—first M2, then M1—was obtained to ensure vascular safety in case of arterial injury.

### 3:31 Tumor Resection.

After extracapsular dissection, the tumor was internally debulked with bipolar coagulation and ultrasonic aspiration, including removal of calcifications. The capsule was progressively mobilized with brain invasion along the medial basal frontal and anterior medial temporal lobes, and carefully excised. The middle fossa component was detached from the temporal lobe and floor, exposing the anterior temporal lobe, uncus, and tentorial incisura. Anteriorly, the tumor was rolled off the brain and dura to expose the optic nerve, while the opticocarotid cistern was dissected from the A1, carotid terminus, and proximal M1. With supraclinoid ICA and proximal M1 heavily involved, meticulous microdissection and debulking were required through the opticocarotid triangle and carotid-oculomotor cistern.

Two long perforators arising from the supraclinoid ICA, just proximal to the bifurcation, were encased by the tumor and carefully dissected free and preserved. During this dissection, the PComA and the AChA were identified. While attempting to dissect the AChA, excessive traction caused a tear in the origin of the artery. This was not repairable with low-intensity bipolar coagulation or microsuturing. Bleeding was stopped by first using bipolar coagulation to assess the extent of injury. We then quickly removed more surrounding meningioma to improve the exposure of the choroidal artery. The anterior choroidal was then exposed from the injury point to the distal point. At this stage, there were no changes in MEPs and SSEPs. We then performed indocyanine green video angiography to assess retrograde filling of the choroidal (we call this the surrogate intraoperative balloon test occlusion), which confirmed that there was an immediate retrograde filling of the AChA, including a small branch coming off from the anterior choroidal.^6^ We then ran MEPs every 5 minutes for a duration of 1 hour and observed no changes in neurophysiological monitoring. During this time, we also induced mild hypotension, something we call "hypotensive challenge," which did not cause any changes in monitoring.

Since this was assuring, alternative strategies were not taken. However, we had entertained these potential options: 1) performing an end-to-end anastomosis, which would be very difficult due to the gap between the 2 ends, 2) reimplanting the anterior choroidal to the ICA or PComA, or 3) a superficial temporal artery–to–anterior choroidal bypass.

AChA injury is potentially catastrophic, so extreme caution is required. Although rare reports describe sacrifice without deficit, this is exceptional.^7^ The remainder of the tumor was detached from the PComA and cranial nerve III. After complete resection, the anterior clinoid dura was removed, and hemostasis was achieved with bipolar cautery, Surgicel, and topical nitroprusside.

### 6:26 Postoperative Imaging and Course.

The postoperative course was uneventful; the patient awoke neurologically intact. MRI showed gross-total resection without diffusion changes. Histopathology confirmed a WHO grade 1 meningioma, and 3-month follow-up MRI revealed no residual or recurrent tumor.

### 6:38 Case 2 Presentation.

The second case is a 41-year-old woman with a 3-year history of intermittent dizziness and a normal ophthalmological exam, who was found to have a large right anterior clinoidal meningioma and referred for management.

### 6:47 Preoperative Imaging.

MRI showed a 3.7 × 3.8 × 4–cm tumor with distal ICA engulfment and temporal lobe/hippocampal mass effect with edema. CTA confirmed distal ICA and proximal M1 involvement, mild inferior MCA narrowing with preserved flow, and PComA abutment.

In this case, the primary surgical objectives were to relieve mass effect, minimize neurological morbidity, and achieve gross-total resection. This patient underwent a pterional craniotomy with extradural anterior clinoidectomy, as done for case 1.

### 7:14 Dural Opening and Arachnoid Dissection.

The dura was opened and reflected anteriorly. The arachnoid over the proximal sylvian fissure was dissected, exposing the M2 branches on the tumor surface.

### 7:22 Tumor Resection.

Early extracapsular planes were established, and the tumor was internally debulked with an ultrasonic aspirator. Attention then turned to the anterior fossa, carefully dissecting MCA bifurcation branches to preserve vascular integrity. The tumor was disconnected from the frontal lobe and rolled laterally to expose the ipsilateral optic nerve, chiasm, and proximal ICA for safe continuation of the vascular dissection. The middle fossa component was detached from the temporal lobe and floor, extending to the tentorial incisura to identify cranial nerve III and the PCA.

The ICA was dissected as far as its terminus, and the MCA was followed retrograde from its distal branches to the bifurcation and along the M1 segment. At the level of the ICA bifurcation, several perforating branches were identified. Dissection proceeded with extreme caution due to the possible involvement of the AChA. This region represented the densest tumor adherence, requiring painstaking and meticulous dissection to protect critical vascular structures.

Additional piecemeal removal improved visualization, enabling identification of the distal end of the AChA. Dissection proceeded very cautiously until the artery was completely freed. The PComA was identified and preserved, followed by complete preservation of all the perforating branches coming off the PCom and AChAs. The tumor was then freed from the skull base and intracranial ICA, and any remaining dural attachments were coagulated and excised.

### 9:32 Postoperative Imaging and Course.

The postoperative course was uneventful; the patient awoke neurologically intact. MRI showed gross-total resection. Histopathology confirmed a WHO grade 1 meningioma, and a 3-month follow-up MRI showed no residual or recurrent tumor.

### 9:44 Conclusion.

Safe management of anterior clinoidal meningiomas with vascular involvement requires meticulous preoperative planning, stepwise tumor debulking, and preservation of arachnoid planes. Continuous neuromonitoring and intraoperative indocyanine green angiography enhance safety by providing real-time assessment of neurological function and vessel patency. Strict adherence to microsurgical principles and careful tumor mobilization are essential to maximize resection while minimizing morbidity in this high-risk group. In the event of arterial injury, surgical teams should be well prepared for backup plans, such as direct arterial repair, in situ bypass, and EC-IC bypass options.

## Disclosures

The authors report no conflict of interest concerning the materials or methods used in this study or the findings specified in this publication.

## Author Contributions

Primary surgeon: Baskaya. Editing and drafting the video and abstract: Sulaimanov, Erginoglu, Keles, Serikkanov, Vera Figueroa, Sevgi, Ozturk. Critically revising the work: Baskaya, Sulaimanov, Erginoglu, Keles, Serikkanov, Sevgi, Ozturk. Reviewed submitted version of the work: Baskaya, Sulaimanov, Erginoglu, Keles, Serikkanov, Sevgi, Ozturk. Approved the final version of the work on behalf of all authors: Baskaya. Supervision: Baskaya.

## Supplemental Information

### Patient Informed Consent

The necessary patient informed consent was obtained in this study.
